# Treatment of Gingival Growth Due to Amlodipine Use With a 445-nm Diode Laser: A Case Report

**DOI:** 10.7759/cureus.32592

**Published:** 2022-12-16

**Authors:** Ömer Faruk Okumuş

**Affiliations:** 1 Department of Periodontology, Erzincan University/Faculty of Dentistry, Erzincan, TUR

**Keywords:** calcium channel blockers, gingival hyperplasia, blue light diode laser, amlodipine induced hyperplasia, gingival overgrowth, gum overgrowth

## Abstract

Amlodipine is a widely used calcium channel blocker associated with gingival enlargement. The effects of amlodipine on gingival enlargement vary depending on the duration of drug use and the dose of the active substance. This report presents a 56-year-old male hypertensive patient who had been using amlodipine (5 mg/day orally, single dose) for the last two years. He presented with diffuse gingival enlargement, complaining of gingival swelling and bleeding. This case report demonstrates the treatment of gingival enlargement with a novel 445-nanometer (nm) blue light diode laser after drug change and oral hygiene, which resulted in permanent and satisfactory clinical results.

## Introduction

Gingival enlargement is caused by many local and systemic factors. As a result of gingival enlargement, suitable areas for the accumulation of dental plaque are created, which can lead to inflammatory periodontal diseases. In addition, gingival enlargement causes an aesthetically undesirable appearance. Various drugs have been implicated in gingival enlargement; for example, anticonvulsants, calcium channel blockers, and immunosuppressants are three drug groups known to cause gingival enlargement [1]. Amlodipine, a calcium channel blocker, rarely causes gingival enlargement [2]. When it does occur, the enlarged gingiva should be surgically removed after changing the drug. Although scalpel surgery is the most common surgical procedure, electrosurgery, radiosurgery, and laser devices of different wavelengths can also be used. A blue light diode laser with a wavelength of 445 nanometer (nm) could be used for this purpose. It is more efficient and safer for surrounding tissues compared with other types of diode lasers with high wavelengths [3].

## Case presentation

A 56-year-old male patient was referred to the Periodontology Clinic of Erzincan University/Faculty of Dentistry, complaining that his gingival tissue had been growing for the past year and that he had experienced increased gingival bleeding for the last seven days. The patient’s anamnesis revealed that he had hypertension and had been taking amlodipine (Norvasc 5 mg tablet) for the past two years.

**Figure 1:**
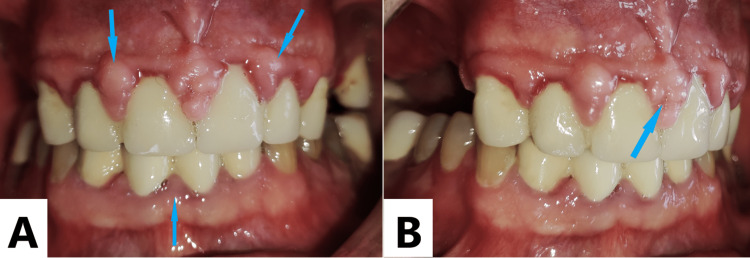
Initial intraoral view. Nodular gingival enlargements (blue arrows) were observed around the metal-supported porcelain-restored teeth. However, gingival enlargement was not observed in teeth without metal-supported porcelain restoration (anterior intraoral view (A), right oblique intraoral view (B)).

During the intraoral examination, it was observed that a metal-supported porcelain restoration had been applied to some teeth, and the patient was found to use removable partial dentures on the lower and upper jaws for the remaining edentulous spaces. Bleeding was observed spontaneously and during probing, and his oral hygiene was observed to be inadequate. Painless, bleeding on probing, and erythematous surface nodular growths were observed around the porcelain-restored teeth in the anterior region of the mandible and maxilla (Figure 1).

In the radiographic examination, horizontal bone loss of 2-3 mm was observed radiographically in the upper and lower anterior teeth. Thus, the patient was determined to have Stage 2, Grade B periodontitis.

First, the patient was referred to his physician, and his medication was changed to an angiotensin II receptor antagonist (Diovan 80 mg film-coated tablet). Initial periodontal treatment, including scaling and root planing using both ultrasonic instruments and universal curettes, was administered along with oral hygiene training. At the end of the first month, the patient’s inflammatory gingival findings had improved; however, no perceptible shrinkage was observed in the hypertrophic gingiva. A diode laser gingivectomy was planned to remove the gingiva that was enlarged. After local anesthesia, the hyperplastic gingival tissue was excised with a semiconductor diode laser (SiroLaser Blue, Dentsply Sirona, Bensheim, Germany) at a wavelength of 445 nm. The bleeding was minimal after the procedure. The patient was prescribed a mouthwash containing chlorhexidine gluconate (Kloroben®, Benzydamine Hydrochloride + Chlorhexidine Gluconate; Drogsan İlaçları Sanayi ve Ticaret Inc., Ankara, Turkey).

In the third week after the operation, the patient’s gingival tissue was observed to have healed; however, marginal gingival erythema was observed around the fixed prosthetic restoration due to insufficient post-surgery oral care. Oral hygiene education was provided to the patient once again. An interface brush was included in his hygiene procedure to clean the gapped interfaces between the restoration and gingiva after healing.

**Figure 2:**
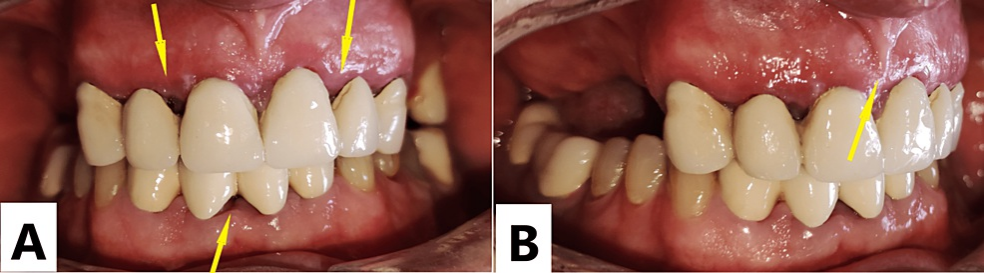
Intraoral view one year after treatment. Gingival enlargement had not recurred (yellow arrows) after one year (anterior intraoral view (A), right oblique intraoral view (B)).

One year later, the patient no longer had any gum complaints, and gingival enlargement had not recurred (Figure 2).

Furthermore, the patient was referred for prosthetic treatment to restore the incompatible restorations. 

## Discussion

Hypertension, cardiac arrhythmia, angina pectoris, and coronary artery spasm are cardiovascular diseases observed in the population, especially in middle-aged and older individuals. Calcium channel blockers are a preferred option for the treatment of such diseases. Amlodipine, a frequently prescribed calcium channel blocker, is a third-generation dihydropyridine calcium antagonist.

Like some other antihypertensives in this group of drugs, amlodipine has been implicated in gingival tissue hypertrophy. The effects of these drugs on gingival enlargement vary depending on the type and dose of the active substance. For example, while 10% of patients using nifedipine develop gingival enlargement [2], the rate is only 1.7%-3.3% in patients using amlodipine [4,5]. Furthermore, gingival enlargement is known to develop faster and more frequently in patients taking 10 mg of amlodipine daily than in patients taking 5 mg [5,6].

Amlodipine-induced gingival enlargement usually occurs within the first three months after starting drug therapy at a dose of 10 mg/day and begins as an enlargement of the interdental papilla [2]. However, according to the results of Jorgensen [5], gingival enlargement was not induced even after six months in patients using 5 mg of amlodipine daily compared with their control group. Contrary to Jorgensen's findings [5], other case reports have reported that 5 mg of amlodipine causes gingival enlargement [7,8].

Calcium channel blockers have been found to inhibit intracellular calcium ion uptake, thereby stimulating gingival fibroblasts. Gingival enlargement does not develop in all patients who take the same drug. The likely cause is that such individuals have fibroblasts that are abnormally sensitive to the drug [9]. In addition, inflammation in the gingivae may influence the relationship between drug and fibroblast activity [9].

In particular, areas of additional plaque retention of mismatched crown-bridge restorations cause more intense gingival inflammation. These areas have an effect on drug-induced gingival enlargement. In the present case, the patient’s erythematous surface nodular growths were located around the bridge restoration, not around his natural teeth.

Notably, the relationship between inflammation in periodontal tissues and gingival enlargement is bidirectional. Hypertrophic gingival tissue makes it difficult for the patient to clean their teeth. Both the increased sulcus depth and the irregular and swollen gingival margin predispose the patient to periodontal disease. Gingival hypertrophy must be treated to maintain adequate oral hygiene and control periodontal inflammation.

Changing the active drug is a critical point in the treatment of drug-induced gingival enlargement [10]. However, initial periodontal treatment with the replacement of the active drug alone is often not sufficient for eliminating gingival hypertrophy [11]. Through the initial periodontal treatment, signs of periodontal inflammation that had been added to the current situation are eliminated [12]. Due to a reduction in bleeding, edema, and redness, although the gingival enlargement continues, some patients may think that adequate healing has occurred [13]. Furthermore, in cases with moderate and advanced gingival enlargement, the growing tissues should be surgically removed through gingival excision using a scalpel, electrosurgery, or laser.

A study demonstrated that the use of a laser instead of a scalpel for removing gingival enlargements significantly reduces the recurrence of the disease [13]. Other advantages of laser surgery over scalpel surgery include less discomfort and minimal or no bleeding [14].

In addition to carbon dioxide and neodymium-doped yttrium aluminum garnet lasers, diode lasers with different wavelengths (e.g., 810 nm or 980 nm) have been used for oral soft tissue surgery [15-17]. In addition, a blue light diode laser with a novel wavelength (e.g., 445 nm) might be a preferred option [3].

Until now, therapeutic diode lasers used in dentistry have generally had wavelengths ranging from 810 nm to 980 nm. A study proved that the 445-nm blue light diode laser device that we used for our procedure has a more efficient cutting depth than traditional high-wavelength diode lasers without increasing thermal side effects [3].

Compared with conventional high-wavelength red light diode laser systems, a crucial advantage of blue light lasers is that they penetrate less into the tissue and are less dispersed [3]. Thus, the desired incision is made without causing serious thermal trauma to the adjacent tissues [3,18].

## Conclusions

In conclusion, drug-induced gingival enlargement has negative consequences for both aesthetic and periodontal health. Rarely, 5 mg of amlodipine can cause gingival enlargement. This case report demonstrates that stable results can be obtained using a low-wavelength diode laser. Prior to performing a laser gingivectomy, initial periodontal treatment along with a change of drug is required.
